# Insights on recent approaches in drug discovery strategies and untapped drug targets against drug resistance

**DOI:** 10.1186/s43094-021-00196-5

**Published:** 2021-03-03

**Authors:** Ramalingam Peraman, Sathish Kumar Sure, V. N. Azger Dusthackeer, Naresh Babu Chilamakuru, Padmanabha Reddy Yiragamreddy, Chiranjeevi Pokuri, Vinay Kumar Kutagulla, Santhivardhan Chinni

**Affiliations:** 1 RERDS-CPR, Raghavendra Institute of Pharmaceutical Education and Research (RIPER)-Autonomous, Anantapur, Andhra Pradesh India; 2 ICMR-National Institute of Research in Tuberculosis, Chennai, Tamilnadu India

**Keywords:** Antimicrobial resistance, ESKAPE bacteria, Antievolution drugs, Drug resistance, Genomic-chemical network, Drug targets

## Abstract

**Background:**

Despite the various strategies undertaken in the clinical practice, the mortality rate due to antibiotic-resistant microbes has been markedly increasing worldwide. In addition to multidrug-resistant (MDR) microbes, the “ESKAPE” bacteria are also emerging. Of course, the infection caused by ESKAPE cannot be treated even with lethal doses of antibiotics. Now, the drug resistance is also more prevalent in antiviral, anticancer, antimalarial and antifungal chemotherapies.

**Main body:**

To date, in the literature, the quantum of research reported on the discovery strategies for new antibiotics is remarkable but the milestone is still far away. Considering the need of the updated strategies and drug discovery approaches in the area of drug resistance among researchers, in this communication, we consolidated the insights pertaining to new drug development against drug-resistant microbes. It includes drug discovery void, gene paradox, transposon mutagenesis, vitamin biosynthesis inhibition, use of non-conventional media, host model, target through quorum sensing, genomic-chemical network, synthetic viability to targets, chemical versus biological space, combinational approach, photosensitization, antimicrobial peptides and transcriptome profiling. Furthermore, we optimally briefed about antievolution drugs, nanotheranostics and antimicrobial adjuvants and then followed by twelve selected new feasible drug targets for new drug design against drug resistance. Finally, we have also tabulated the chemical structures of potent molecules against antimicrobial resistance.

**Conclusion:**

It is highly recommended to execute the anti-drug resistance research as integrated approach where both molecular and genetic research needs to be as integrative objective of drug discovery. This is time to accelerate new drug discovery research with advanced genetic approaches instead of conventional blind screening.

## Background

The term “drug resistance,” more commonly referred to medications such as antibiotics, is now extended to anticancer agents. The drug-resistant infectious diseases, including multidrug-resistant (MDR)/ extensively drug-resistant (XDR)-tuberculosis, infection due to methicillin-resistant *Staphylococcus aureus* (MRSA), vancomycin-resistant *Staphylococcus aureus* (VRSA), carbapenem-resistant *Enterobacteriaceae* (CRE), drug-resistant *Clostridium difficile* and drug-resistant cancer are the most life-threatening health issues, which need prioritized focus in research [1, 2]. Recently, in addition to multidrug-resistant (MDR) pathogens, “ESKAPE” (*Enterococcus faecium*, *Staphylococcus aureus*, *Klebsiella pneumoniae*, *Acinetobacter baumannii*, *Pseudomonas aeruginosa* and *Enterobacter species*) bacteria has also emerged which can withstand lethal doses of all antibiotics. The World Health Organization (WHO) predicted that antimicrobial resistance (AMR) is expected to cause 10 million deaths annually by 2050 [3].

### Underlying reasons for drug resistance

In addition to the drug-resistant bacterial and mycobacterial infections, drug-resistant cancer and drug-resistant protozoa, fungal and viral infections are also more prevalent; thus, the hope on successful chemotherapy for these diseases is not optimistic in the near future. The various reasons for the emergence of super bugs include irrational use of antimicrobials/antibiotics, spontaneous mutations in microbes, lack of regulatory control and supervision on antibiotic use, and lack of new antibiotics with novel mechanism of action, reluctance of industries to under research on antimicrobial resistance, frequent exposure of human to disinfectant and inappropriate choice of disinfectants. As per the WHO, British Society for antimicrobial therapy, Centre for Disease Prevention and Control (CDC), National Institute of Health, the collective underlying reasons for the existing serious antimicrobial crisis are (a) lack of new antimicrobials and antibiotics, (b) lack of diverging approach in drug discovery strategy, (c) increasing ineffectiveness of existing antibiotics, (d) increasing mutation of common pathogens, (e) irrational use of antibiotics, and (f) lack of awareness in antibiotic use [4, 5].

Furthermore, the level of drug resistance in microbes is not measurable, but on the basis of resistance levels, the drug-resistant infections can be categorized as multidrug drug-resistant (MDR), extensively drug-resistant (XDR), pan-drug-resistant (PDR) and totally drug-resistant (TDR) infections [6].

Overall, the existing antibiotics used in the treatment of infectious disease since 60 years are now becoming ineffective due to drug resistance. A recent study reported that in the USA, each year at least 2 million people become infected with drug-resistant bacteria, among them 23,000 people die due to antibiotic treatment failure. In fact, the drug resistance is not a new phenomenon observed today. It has been postulated since 1940 with the discovery of penicillin and the resistance produced by *Staphylococcus aureus* to penicillin G (benzyl penicillin). The percentage of drug resistance in developing countries like India is 2–3-fold higher than that of developed countries like the UK and the USA [7].

### Types of antimicrobial resistance (AMR)

**Fig. 1:**
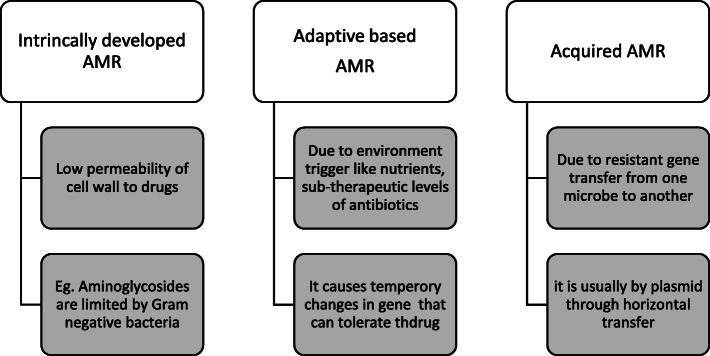
Classification of drug resistance

AMR is classified into different types based on the phenomenon of drug resistance, specificity towards antibiotics and the severity of threats to human health. The phenomenon of development of resistance in microbes includes *intrinsic AMR* (due to low permeability of cell wall to drugs), *adaptive AMR* (environment trigger like nutrients, sub-therapeutic levels of antibiotics) and *acquired* AMR (resistant gene transfer from one microbe to another) [8] as shown in Fig. 1.

Based on the specificity of microbes towards antibiotics, AMR is classified as *antibiotic-specific AMR* and *antibiotic non-specific AMR*. In non-specific AMR, microbes resist the entry of all small molecules into the cell, thereby preventing or decreasing the intracellular concentration of antibiotics. The mechanism of non-specific AMR includes low cell walls permeability of drugs and downregulation or decreased porin proteins in the cell membrane (porin is one of the channel protein and is responsible for the transportation of small polar molecule and ions across the phospholipid bilayer of bacteria). In antibiotic-specific AMR, mutation or region-selective modification of specific target is responsible for drug binding (mutation of bacterial penicillin binding proteins (PBPs) to penicillin drug [9].

**Fig. 2:**
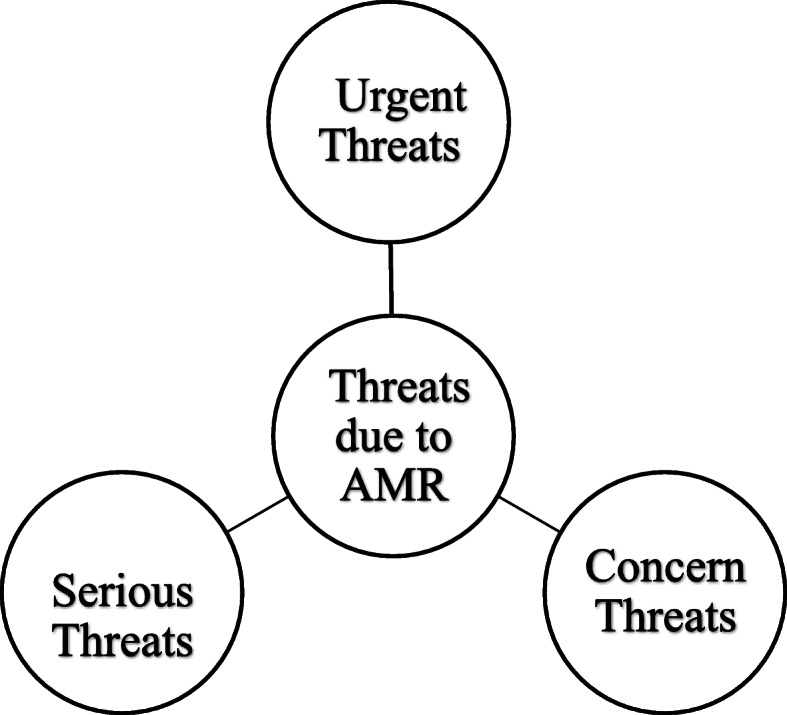
CDC classification of drug resistance based on severity

According to CDC 2019, the drug-resistant microbial species classified based on the severity as urgent, serious and concern threats are shown in Fig. 2 and Table 1. Urgent threats are highly magnitude AMR threats because of the noteworthy risks identified across several conditions.

**Table 1:** Centre for Disease prevention and Control (CDC) classification on drug-resistant infections

Threats	Examples of drug-resistant pathogens that cause public threat
Urgent threats	Drug-resistant *Clostridium difficile*, Carbapenem-resistant *Enterobacteriaceae* (*CRE*), Drug-resistant *Neisseria gonorrhoeae.*
Serious threats	Multidrug-resistant tuberculosis (*MDR-TB*), Drug-resistant Acinetobacter Species Drug-resistant campylobacter Species Drug-resistant *Pseudomonas* Species Drug-resistant *Salmonella*species Drug-resistant *Shigella*species Methicillin-resistant *Staphylococcus aureus (MRSA)* Extended spectrum β-lactamase producing *Enterobacteriaceae* (*ESBLs*), Drug-resistant *Streptococcus pneumonia* Fluconazole-resistant *Candida albicans.*
Concerning threats	Vancomycin-resistant *Staphylococcus aureus* (*VRSA*), Erythromycin-resistant Group A *Streptococcus* Clindamycin-resistant Group B *Streptococcus.*

These threats might not currently be widespread but have the potential to become, so a vital attention is required to limit transmission. Serious threats are due to significant antibiotic resistance; these threats will get worse and might become crucial without fragmentary public health monitoring and anticipation activities. Concerning threats are bacteria for which the risk of AMR is low and/or there are numerous therapeutic options for resistant infections. These bacterial pathogens cause severe illness [10].

**Fig. 3:**
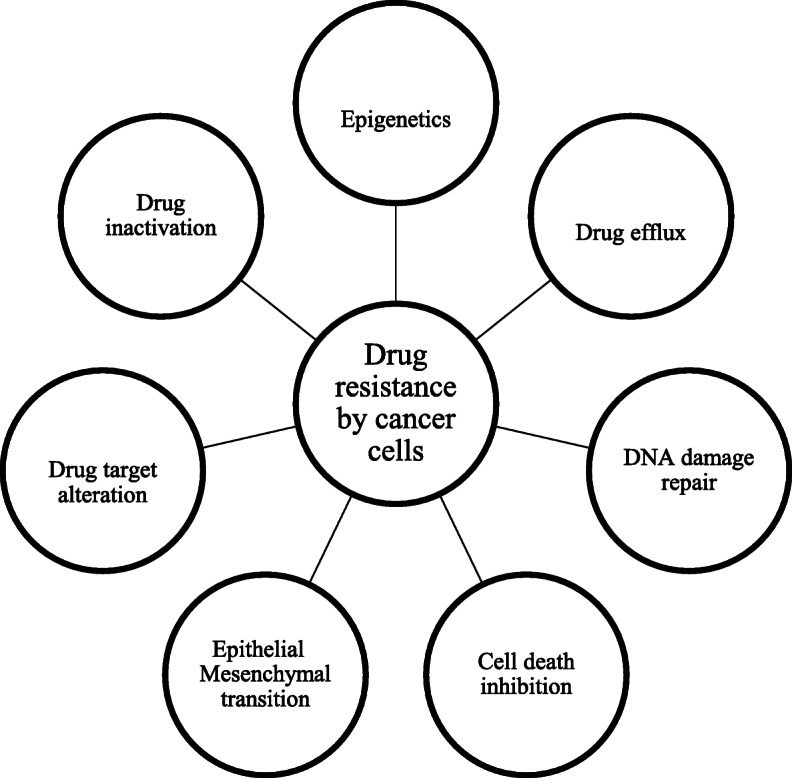
Mechanism of drug resistance in cancer

In the early days, drug resistance was associated with antibiotic treatment, but since one decade, drug resistance has been observed with cancer chemotherapy as well. The phenomena of drug resistance by cancer cells, called as “antineoplastic resistance”. In cancer drug resistance, patients will initially respond to cancer chemotherapeutics, but over time, they do not respond to chemotherapeutic agents due to the development of drug resistance by tumour cells [11] as illustrated in Fig. 3. Interestingly, the underlying mechanisms are very close to antimicrobial resistance.

### AMR as current global epidemics

According to the CDC report 2019, each year about **2.8 million antibiotic-resistant infections occur in USA, resulting in about 35,000 patients died.** In addition, 223,900 cases of *Clostridioides difficile* were also observed among which 12,800 patients died [10]. Recently, the WHO released top 10 global threats to health in 2019, among them AMR to tuberculosis drugs have accounted the huge contribution with 1.6 million deaths per year around 10 million cases [5]. The twist in the drug-resistant microbe story is that it was not the AMR microbes that have spread and are found in remote areas of all the countries. In addition to the above, globally, several drug-resistant infections are in rise including the following: (a) 64% of *Staphylococcus aureus* infections are notified as MRSA infections, (b) multidrug-resistant *Klebsiella pneumoniae* has become the most hospital-acquired infection responsible for pneumonia, bloodstream infections and infections of newborns and intensive-care unit patients, (c) recently carbapenem antibiotics have become ineffective against *Klebsiella pneumoniae* infections, (d) the resistance of *Escherichia coli* to fluoroquinolones has turned as a threat in the treatment of urinary tract infections, (e) treatment failure against gonorrhoea and its resistance to third-generation cephalosporin antibiotics has been documented in countries like Australia, Austria, Canada, France, Japan, Norway, Slovenia, South Africa, Sweden and the UK of Great Britain and northern Ireland [3]. To achieve the Sustainable Development Goals, AMR needs urgent multi-sectoral action (SDGs). AMR’s cost to the economy is important; prolonged disease results in longer hospital stays, the need for costlier medications and financial difficulties for those affected. The effectiveness of modern medicine in treating diseases, even during major surgery and cancer chemotherapy, would be at elevated risk without successful antimicrobials [3].

## Main text

### Molecular mechanisms of drug resistance

#### Drug-resistant bacteria

The spreading of drug resistance is not because the entire bacterial population. Usually, this is due to one in thousands of bacteria that might have acquired a resistant gene through single or multiple de novo mutations. Bacteria develop resistance to antimicrobials through various mechanism including inactivation (by beta-lactamase enzyme), alteration of the target (mutation of penicillin binding protein), circumvention of the target pathway (folate synthesis) or efflux of the antimicrobials [2]. In addition, bacteria can also develop resistance through mutation in the existing genes and/or acquiring resistant genes from other strains or species [6]. The environmental reasons for the bacterial resistance are (a) severe or multiple infections in humans; (b) use of antibiotics in animals/birds which are ingested by patients; (c) incorrect antibiotics in treatment due to lack of awareness or due to inappropriate prescription and (d) inadequate serum level of antibiotics caused by in appropriate dose of antibiotics or poor pharmacokinetics or spurious antibiotics drugs or infection at the elimination phase of prior drug and one half life or patient incompliance to the recommended therapy [12]. The various drug resistance mechanisms for each class of antibiotics are enlisted in Table 2.

**Table 2:** Reported mechanisms of drug resistance for various antibiotics

Class of antibiotics	Mechanisms	Descriptions
Beta-lactam antibiotics *Penicillin*, *Cephalosporin*, *Imepenam.*	a) Enzyme destruction	Destruction of the beta-lactam ring of antibiotic molecule.
	b) Mutation of PBPs	Methicillin resistance is due to the modification at allosteric binding site of PBPs.
	c) Down regulation of porins	Diminished the transportation of polar antibiotics into the bacterial cell. *Pseudomonas aeruginosa*, *Klepsiela pneumonia r*esistant to imepenam
Aminoglycosides *Streptomycin*, *Kanamycin*, *Gentamycin*	a) Ribosomal mutation	Mutation of bacterial A site on 16s RNA of 30S ribosome (Mycobacterium resistance to Streptomycin)
	b) Destruction by aminoglycoside metabolizing enzyme (AME)	There are three major enzymes, AACs (AG N-N acyltransferase), ANTs (AG O – Nucleotidyl transferase, APHs (AG O – phosphotransferase). Among all, AACs are more common in Gram-negative bacteria.
	c) Cell membrane modification	In case of OM modification, the cell membrane is modified by incorporation of positively charged 4-amino-4-deoxy-L-arabinose, which repulses the polycationic aminoglycoside.
	d) Efflux pump	The efflux pump which decreases the intracellular concentration.
Fluoroquinolones *Ciprofloxacin*, *Ofloxacin*, *Levofloxacin*	a) Decreased drug update	It may be due to the alteration in OM and activation of efflux pump. Both are common in Gram-negative bacteria, but S. aureus shows drug resistance through an efflux mechanism alone.
	b) Altered target	The two enzymes, which bind with fluoroquinolones, undergo mutation viz., DNA gyrase (Gram-negative bacteria) and topoisomerase IV (Gram-positive bacteria).
	c) Qnr protein mediated	Qnr protein is due to expression of mutation that protects the nucleic acid enzyme from binding to fluoroquinolones.
Glycopeptides *Vancomycin*	Mutation in cell wall precursor component by replacement of C-terminal D-alanine with D-lactate or D-serine	There are six types of resistance (Van A, Van B, Van C, Van D, Van E and Van G) among these ABDEG are acquired resistance whereas C is intrinsic. Van A and B are located at plasmid where the rest of them located in the chromosome.
Macrolides/lincosamides *Erythromycin*, *Oleondomycin*	a) Target site modification by methylation (Streptococci) at 23s rRNA of 50S ribosome.	There are nearly 40 erm genes are found among them erm A, B, C, F is reported in pathogenic microbes like *Streptococcus*, *Enterococcus* and *Bacteroids*
	b) Efflux pumps	In Gram-negative bacteria, it is mediated by ABC (ATP-binding cassette transporter) and MFS (major facilitator super-family). In case of Gram-negative bacteria, it is mediated by chromosomally encoded pumps.
	c) Drug inactivation of enzymes	The enzyme like esterase and phosphoesterase *(Enterococci*) destroys erythromycin, 14, 15 member macrolides. But these enzymes do not destroy lincosamides.
Sulphonamides *Sulfomethoxazole* *Sulfodoxine* *Sulfodimidine*	Mutation of DHPS enzyme (Dihydropteroate synthase) responsible for binding of sulphonamide.	Mediated by sul1 and sul2 genes, which are mediated by horizontal transfer (plasmid coded). Trimethoprim shows resistance via plasmid borne resistance.
Tetracyclines *Doxycycline*, *Minocycline*, *Glycylcycline*	a) Tetracycline efflux pump (efflux or TET proteins)	Efflux resistant genes are mediated by plasmids. Gram-positive efflux is regulated by an attenuation mechanism whereas Gram-negative efflux is mediated by repressor that binds with tetracycline.
	b) Drug modification	Chemical modification tetracycline by a cytoplasmic protein in presence of NADPH and Oxygen. But still it is unclear.
	c) Target mutation	Modification of 30S ribosome, which is responsible for the attachment of aminoacyl tRNA to RNA ribosome.
	d) By specific ribosome protection protein (Tet (O), a translational GTPase)	There are nine ribosomal protection proteins reported that protect the ribosome from tetracyclines. This is mediated by both plasmid and self-transmissible chromosomal elements (Conjugative transposons).

#### Drug resistance in *Mycobacterium tuberculosis*

The emergence of drug-resistant behaviour has been reported in the *Mycobacterium tuberculosis H*_*37*_*R*^*1*^ (mtb) strain (WHO 2016). Now, there are many clinically diagnosed drug-resistant forms of tuberculosis (DR-TB) including (1) isoniazid-resistant TB; (2) rifampicin-resistant TB; (3) totally drug-resistant tuberculosis (TDR-TB or XXDR-TB); (4) multidrug-resistant tuberculosis (MDR-TB); (5) extensively drug-resistant tuberculosis (XDR-TB); (6) mono-drug-resistant tuberculosis and (7) poly-drug-resistant tuberculosis. The abovementioned variants of TB are difficult to treat even with duration over 20 months. Despite the initiatives of TB control strategies, it seems more than 30% of the TB infections cases are beyond the incurable XDR stage. According to the WHO 2017, India has the highest incidence of TDR/MDR/XDR-TB cases (> 80%), the TB crisis is likely to get worse and 12.4% of the patient population in India will have a variant of TB by 2025 [13]. The cost and success rate for treating drug-resistant TB are mentioned in Table 3.

**Table 3:** Comparative drug treatment schedule for data of TB/MDR/XDR-TB—cost, duration and success rate

Drug/treatment parameter	Drug susceptible tuberculosis	Drug-resistant tuberculosis		
		MDR-TB	XDR-TB	TDR-TB
Isoniazid	Yes	No	No	No
Rifampin	Yes	No	No	No
Fluoroquinolones	Yes	Yes	No	No
Injectable	Yes	Yes	No	No
First line treatment	No	No	Yes	No
Second-line treatment	Yes	No	No	No
Duration of treatment	6 months	2 years	2 years	> 2 years
Cost of therapy	50 $	5000 $	50,000$	> 100,000 $
Percentage rate of cure	90 %	50 %	10 %	0–1 %

Overall, the future prediction on the treatment of drug-resistant TB epidemic is not optimistic [14]. Recently introduced new TB agents such as delamanid, bedaquiline and pretomanid demonstrated the dissatisfactory level of efficacy against drug-resistant TB and are very toxic as well [15, 16]. Recently in the year 2020, 2-ethylthio-4-methylaminoquinazoline has been reported as inhibitor of cytochrome bc1 for TDR/MDR/XDR-TB [17]. The Mycobacterium species elicit drug resistance via both intrinsic and acquired mechanisms [15].

Intrinsic drug resistance is due to the unusual structure of mycolic acid cell wall. This unusual structure causes low permeability of antitubercular drugs through efflux mechanisms especially to tetracycline, fluoroquinolones and aminoglycosides. *Mycobacterium smegmatis* showed the lack of porin MspA, which accounted for 10-fold increase of lethal dose for many drugs including ampicillin, cefaloridine, vancomycin, erythromycin and rifampicin. In addition, beta-lactamase enzyme also degrades lactam antibiotics [18]. In *M. tuberculosis*, the function of gene Rv1698 is same as MspA and attributing the resistance to hydrophilic drugs. Both Rv1698 and Rv1973 are serving as a mycobacterial outer membrane protein (OMP) and they are responsible for intrinsic resistance. The physiological adaptation due to MDR determinants also accounted for intrinsic drug resistance [19].

The mycobacterial acquired resistance occurs due to the spontaneous mutations of chromosomal genes that is taking place during sub-optimal drug therapy. The responsible genes for mutations are katG, inhA, ahpC (Isoniazid), rpoB (rifampicin), pncA (Pyrazinamide), rpsL, rrs, gidB (Streptomycin), embB (Ethambutol), gyrA/gyrB (Fluoroquinolone), rrs (Kanamycin and Amikacin), tlyA (Capromycin and viomycin), ethA (Ethionamide), thyA (p-aminosalicylic acid), Rv3547 (Delamanid, PA-824 and OPC-67683) and atpE (TMC207) [20, 21]. The mechanism/role of these genes are shown in Table 4.

**Table 4:** Mechanism/role of mycobacterial genes responsible for resistance

Genes	Mechanism/role
KatG	Catalase/peroxidase of isoniazid
inhA	Covalent attachment of INH – NAD by enoyl reductase
ahpC	Mutation to ahpC gene causes over production of alkyl hydroperoxide reductase
rpoB	Crucial enzyme (RNA polymerase) in the transcriptional process
pncA	Deamidation of nicotinamide (NAM) into nicotinate
rpsL, rrs, gidB	S12 ribosomal protein, 16S rRNA, 7-methyl guanosine methyltransferase—inhibits protein synthesis
embB	Polymerization of arabinogalactan
gyrA, gyrB	Catalyses the ATP-dependent negative super-coiling of double-stranded closed-circular DNA
rrs, tlyA	16S rRNA, rRNA methyltransferase—ribosome biogenesis and translation
ethA	Catalyse the terminal reaction in the fatty acid elongation cycle
thyA	Plays a key role in the biosynthesis of thymidylate
Rv3547	Activation of the drug (Delamanid)
atpE	Encodes the c part of the F0 subunit of the ATP synthase

#### Drug resistance in fungi

**Fig. 4:**
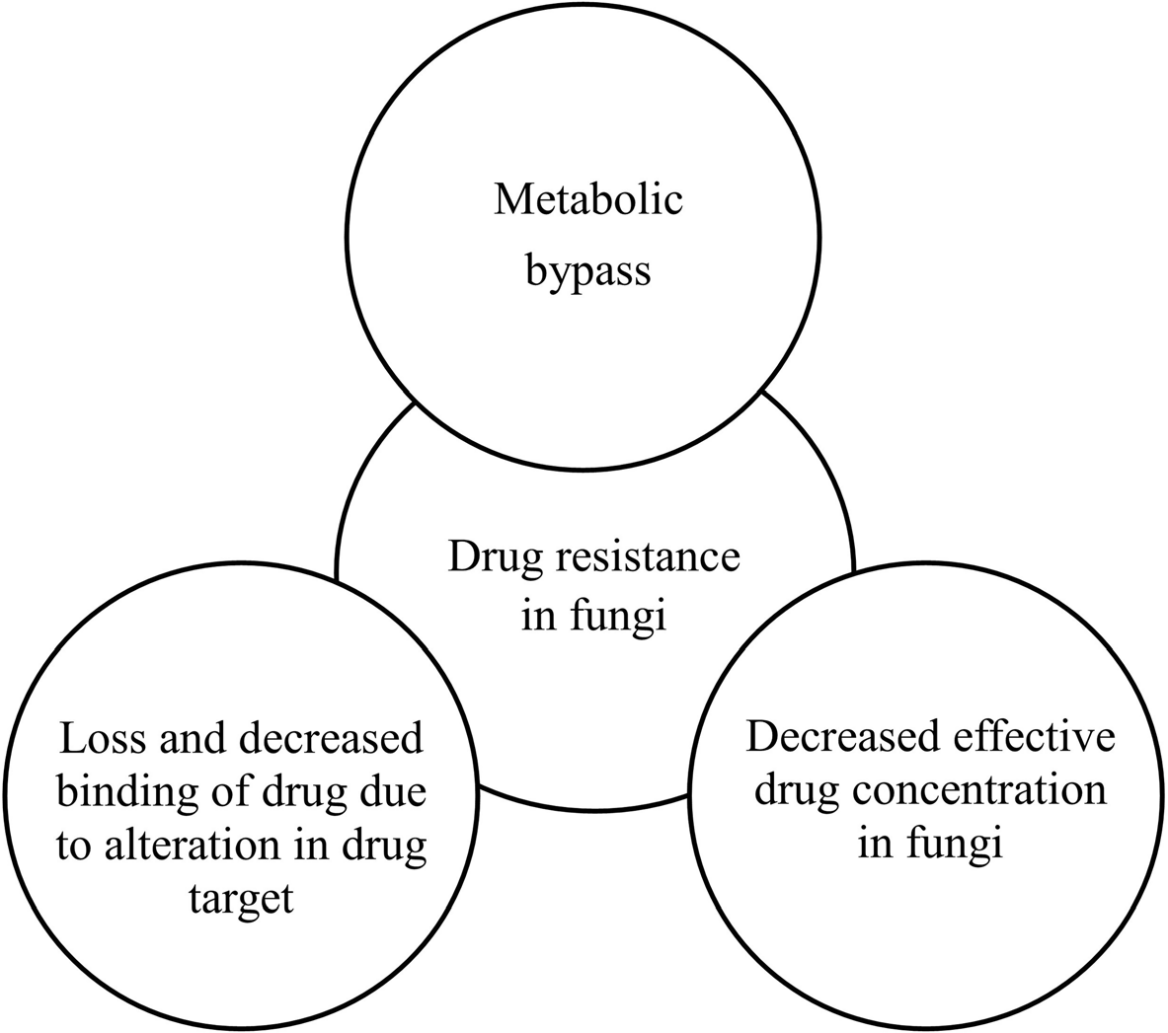
Drug resistance mechanism in fungi

Fungal diseases caused by diverse pathogens, including *Candida*, *Aspergillus*, *Pneumocystis* and *Cryptococcus sp*., have also developed resistance to all antifungal drugs. Among all, *Candida* species are ranked as the most virulent fungi and fourth most common microbes of life-threatening bloodstream infections after bacterial pathogens [22, 23]. Thus, the mortality rate due to Candidemia is very high (~ 50%). Next, the *Aspergillus* infections are reported in hematopoietic stem cell transplant (HSCT) recipients with about 30–50% death [24]. The various mechanisms of drug resistance exerted by fungi are elucidated in Fig. 4. These mechanisms include:

##### Active efflux pump or transport

The decreased intracellular drug concentration of antifungal agents is mediated by efflux transport systems of fungi, such as ATP-binding cassette (ABC) transporters and major facilitator super-family (MFS) transporters [25]. The genomic analysis revealed the different topologies responsible for resistance that vary from species to species. For example, *Candida albicans* contains 28 ABC proteins and 96 potential MFS transporters but *Candida glabrata* contains 18 ABC transporters and 33 MFS transporters [26]. The upregulation of the gene *ERG11* is responsible for the fluconazole resistance in *C. albicans* [27] *whereas* upregulation of *Cyp51A* is responsible for fluconazole-resistant *Aspergillus fumigatus* isolates [28].

##### Drug target alterations

This mechanism has been notified in fungi against azoles and echinocandins. The respective target for these two drugs are a 14α-lanosterol demethylase and a β-1,3 glucan synthase. Among these, Lanosterol demethylase is encoded by *ERG11* (*C. albicans*) and *Cyp51A* and *Cyp51B* (*A. fumigates*) [29]. The occurrence of mutations in *ERG11* resulted in non-synonymous amino acid substitutions (azole-resistant *C. albicans*), which accounted for the decreased affinity of the target to azoles. In case of echinocandins resistance, β-1, 3glucan synthases are encoded by *FKS* genes [30].

##### Metabolic bypasses

These are very common compensatory mechanisms in all microbes, where microbes divert the toxic effect of antifungal drugs. For example, fluconazole resistance in fungi is mediated through the loss-of-function mutations in the gene *ERG3* which encodes a sterol Δ^5,6^ desaturase. It catalyses the introduction of a C=C double bond in the ergosta-7,22-dienol and gives ergosterol—a final step in the biosynthesis of ergosterol [31]. In the active state, 14 α-methylated sterols (arise from azole exposure) are converted to 3,6-diol derivative. Therefore, if the fungus acquires azole resistance, it cannot produce the metabolite [32].

#### Drug resistance in malarial parasites

Malaria is a deadly parasitic disease occurring in humans, caused by species of Plasmodium Protozoan namely *P. falciparum*, *P. vivax*, *P. malariae*, *P. ovale* and *P. knowlesi*. Among the species, *Plasmodium falciparum* is the most virulent parasite documented for high mortality. Every year, it kills about 2 million people, especially children. Drug resistance in malarial parasite (antimalarial drug resistance) is against all antimalarial drugs including artemisinin (artemisinin-resistant malaria) [33, 34]. However, the de novo emergence of resistance is now treated with artemisinin combinational therapies (ACTs) with other malarial drugs [35].

*The genetic basis of antimalarial drug resistance is rare*, *but it is spontaneous and independent of the type of antimalarial drugs. The de novo mutation of P. falciparum is single or multiple. There was a report that chloroquine-resistant P. falciparum* is mutagenic, which occurred due to the mutation in a gene encoding a transporter—*P. falciparum chloroquine-resistant transporter (Pf*CRT). Once the mutation occurs at *Pf*CRT, the subsequent mutation occurs in the second transporter (*Pf*MDR1) and this is responsible for the modulation of the level of resistance. Nevertheless, the significant role of *Pf*MDR1 mutation in therapeutic response of chloroquine treatment is unknown [35, 36].

Another gene Pfnhe1 found in *P. falciparum*, coded for sodium hydrogen exchanger (Na^+^/H^+^ exchanger or PfNHE), is associated with quinine resistance. The bi-functional dihydrofolate reductase-thymidylate synthase from *P. falciparum* (PfDHFR-TS) located on chromosome 4 for pfDHFR responsible for pyrimethamine resistance. Cytochrome b (Cytb) gene is a subunit of cytochrome bc1 complex is responsible for binding of atovaquone at ubiquinol site. The mutation of this gene is responsible for atovaquone resistance. Artemisinin resistance is due to the single-nucleotide polymorphisms; *Plasmodium*’s K13 gene accounted for unfolded protein response pathway. This antagonizes the pro-oxidant activity of artemisinin in parasites [37, 38]. The WHO already banned the oral artemisinin-based monotherapy (oAMT) which develops and spreads the drug resistance to artemisinins [39].

#### Drug resistance in virus

The viral replication biology is very critical not only for understanding the evolution of virus towards drug resistance, but also for developing new antiviral drugs. The drug resistance in virus has been well documented including for hepatitis C, influenza A virus (IAV), herpes simplex virus (HSV), human cytomegalovirus (HCMV), retrovirus HIV and hepatitis B virus (HBV) [40].

Hepatitis C virus (HCV) is well known for its mutation rate and high genomic diversity and is facilitated by its frequent replication and very low proofreading function of viral encoded RNA polymerase. The direct-acting antiviral drug (DAA) usually inhibits either protease or polymerase activity. The drug which inhibits protease possess low genetic barrier to resistance, it means that resistance develops very easily through one or few mutations. To overcome this HCV resistance, the combinations of the DAA agents (ledipasvir and sofosbuvir) are recommended and these combinations exhibit higher genetic barrier to resistance with very less cross-resistance between the two drugs [41].

IAV is found with small genome codes with 11 proteins including its two surface antigen proteins namely hemagglutinin (HA) and neuraminidase (NA). These surface antigens, HA and NA, evolve relatively more rapid and higher than any other viral proteins. The current drugs used to treat IAV infections are NA inhibitors and they inhibit the detachment of viral envelope from the cell membrane. The most popular NA inhibitor oseltamivir has found to be associated with the mutation of H274Y and confers the high level of resistance. The drug zanamivir has not been observed to develop resistance, but is not recommended for frequent administration, this may also have contributed to the rarity of resistance. The latest antiviral drug called favipiravir acts by inducing mutagenesis in the IAV and the level of resistance to favipiravir is yet to be documented [42].

HSV contains a large genome with low diversity as compared with RNA viruses. Usually, the systemic antiviral therapy is needed only in immune-compromised patients. The common drugs are acyclovir and its derivatives and all nucleoside inhibitors. For these agents, resistance mutations are well known, they affect either thymidine kinase or DNA polymerase [43].

The genome of HCMV is diverse within-host and exhibits different levels of polymorphism when compared to RNA viruses, even though DNA polymerase possesses a higher rate of fidelity than RNA virus polymerases [44]. The treatment for HCMV infections usually involves the use of nucleoside analogues such as ganciclovir and cidofovir. Resistance develops either in a viral kinase responsible for phosphorylation or in the DNA polymerase [44].

HIV is a retrovirus encoding an RNA genome within the virion, but it replicates its genome using reverse transcriptase and generates a copy of DNA and eventually double-stranded DNA. During the drug treatment, the viral reverse transcriptase is relatively more prone to error and shows a high rate of nucleotide substitutions, increased population diversity and frequent resistance mutations. The HIV therapy today is a multiple drug regimen consisting of nucleoside reverse-transcriptase inhibitors, non-nucleoside reverse-transcriptase inhibitors, protease inhibitors and/or integrase inhibitors [45].

HBV is an enveloped DNA virus, and it transcript to RNA intermediate and then again reverse transcribed to DNA; furthermore, the HBV exists as a quasi-species with high levels of diversity through very poor proofreading during reverse transcription phase. Therefore, like RNA viruses, HBV infections preserve polymorphism at all nucleotide positions within a host, thus providing the way for resistance mutations to fight against drugs. HBV is commonly treated with reverse-transcriptase inhibitors, particularly lamivudine [46].

#### Drug resistance in tumour cells

In the early days, drug resistance was associated with antibiotic treatment, but since one decade, drug resistance has been observed with cancer chemotherapy as well. The phenomena of drug resistance by cancer cells is called “antineoplastic resistance”. In cancer drug resistance, patients will initially respond to cancer chemotherapeutics, but over time, they do not respond to chemotherapeutic agents due to the development of drug resistance by tumour cells [11] as illustrated in Fig. 3. Interestingly, the underlying mechanisms are very close to antimicrobial resistance.

**Fig. 5:**
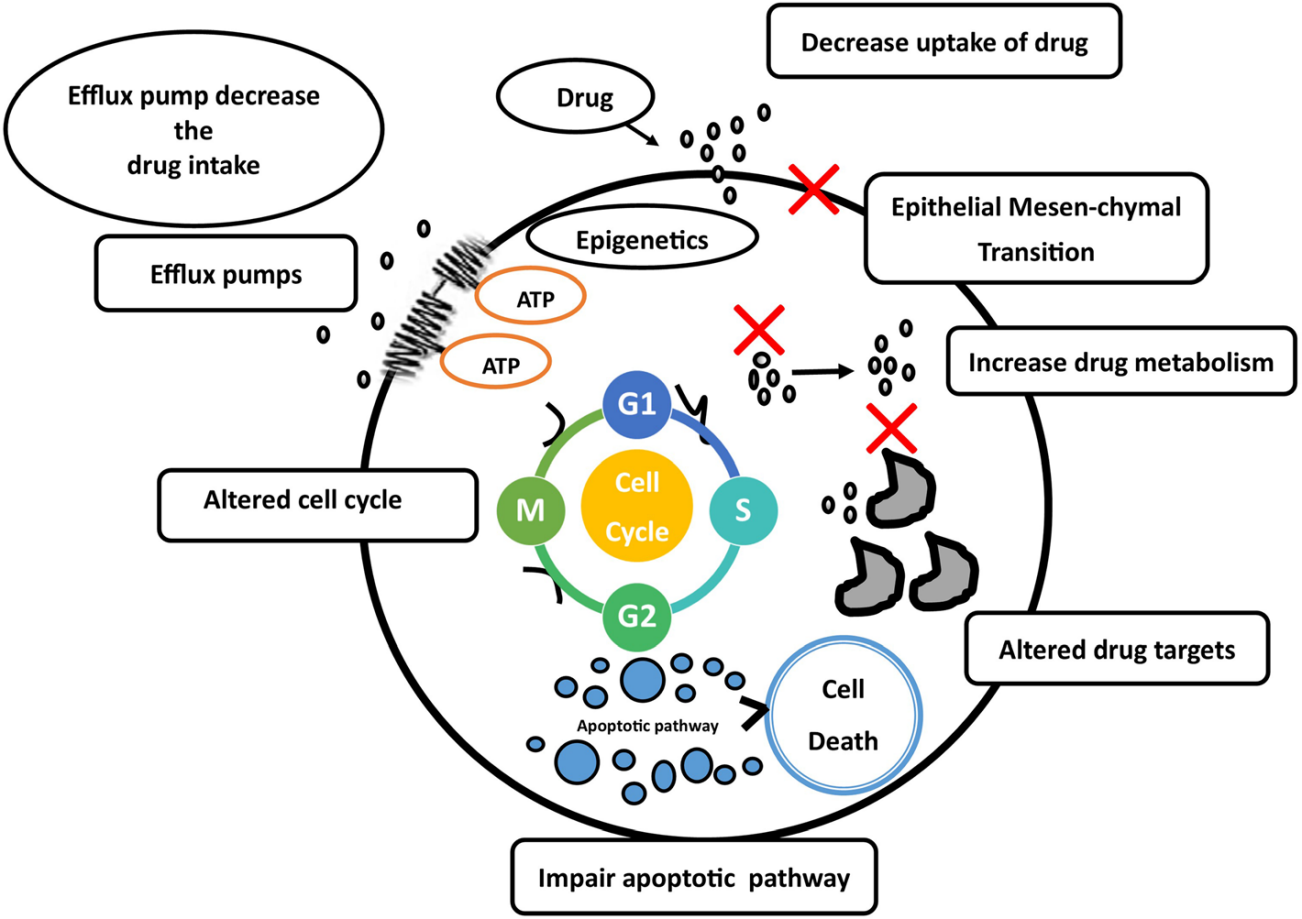
Various mechanisms of drug resistance in cancer

The distinct feature of resistance is usually a tumour cell survives and relapses during and after chemotherapy by a variety of intracellular mechanisms acquired by mutations. Various intracellular mechanisms exerted by resistant tumour cell are *altered drug metabolism* (increased drug efflux, decreased drug intake, enhanced drug detoxification, sequestration), *modification of a drug target*, *dysregulation of apoptotic protein* and *enhanced DNA repair* [37]. This antineoplastic resistance occurs due to DNA mutations of tumour cells, and they are (a) DNA-synthesis gene over expression (against anti-metabolite drugs); (b) altered target molecules (against tyrosine kinase antagonists); (c) enzyme deactivation; (d) altered membrane transport; (e) enhanced DNA repair—ERCC1 (against platinum-based drugs); (f) resistance to drug-induced cell cycle arrest and (g) resistance to apoptosis [38]. The above mechanisms are shown in Fig. 5.

### Recent development in drug discovery against drug resistance

#### Policies on discovery of new drugs against drug resistance

Developed countries have already accelerated the discovery of new sterile antibiotic molecules for the treatment drug-resistant infectious diseases including MDR/XDR-TB. The reflected recommendations are (a) to support more basic research relevant to antimicrobial drug development; (b) to support the study of virulence factors, host–pathogen interactions and genome mining; (c) to discover newer agents with new mechanisms of action and (d) to exploit the new natural and biotechnological products in antimicrobial drug discovery [3, 4].

As a result, there were many new antibiotic molecules developed, but were precluded from further development due to various reasons including the following: (1) the newer antibiotics are structurally similar to existing ones, so resistance is not restricted; (2) all targets were highly susceptible for mutations (MurA, PBP2); (3) spontaneous resistant mechanisms of microbes are not predictable; (4) genome variation in targets from strain to strain; (5) cytotoxicity; (6) carcinogenicity; (7) non-selectivity of highly potent molecules; (8) poor drugable property. As per the existing reports, more than 90% of the developed hit molecules were declared as not suitable for drug-resistant infections, whilst the rest of them were low potent against super bugs or have high cytotoxicity in humans (*including Oritavancin and Dalvance*). Therefore, the WHO declared this era as the antibiotic crisis era, it means there is an urgent need for new agents and new strategy to save millions of lives from antimicrobial resistance [5, 7].

#### Drug discovery void

**Fig. 6:**
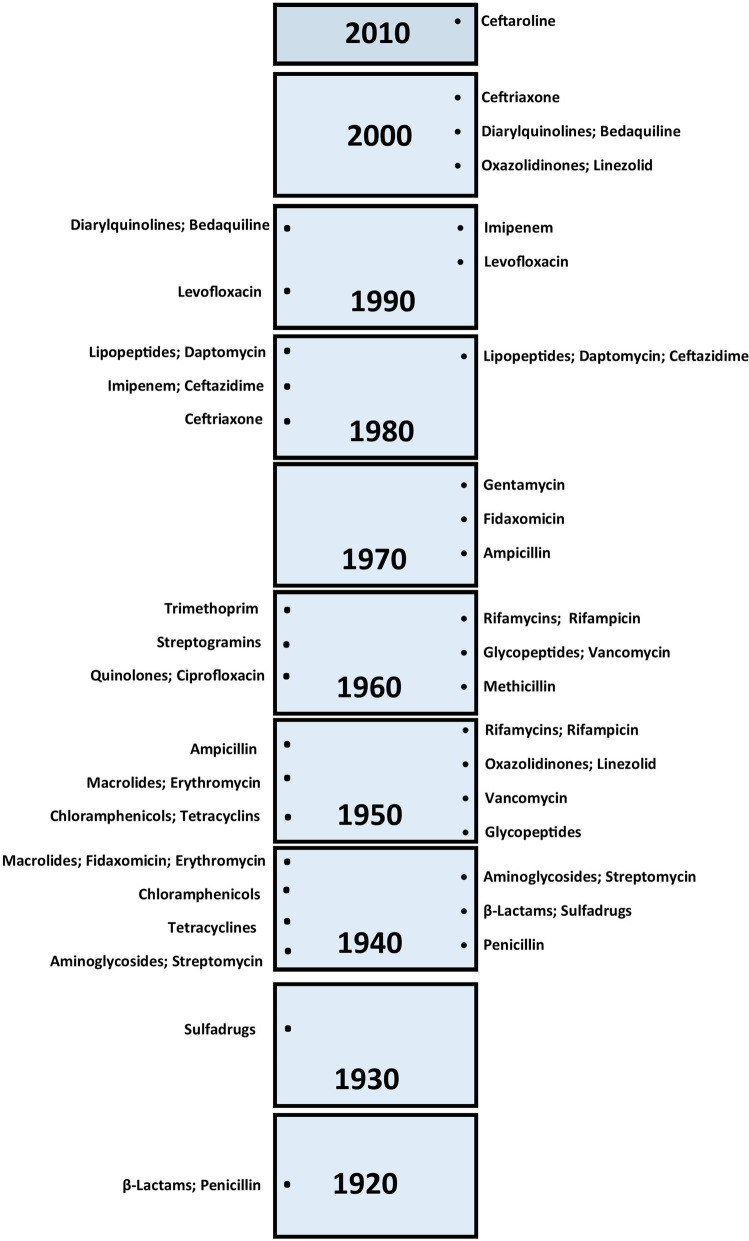
Timeline of various antibiotics discovery (left) and resistance observed (right)

With the review of the time scale of the discovery of new antibiotics, drug resistance was observed with the discovery of penicillin itself. The discovery of beta-lactamase inhibitors such as clavulanic acid and sulbactam is an outcome of research to fight against drug resistance. After the introduction methicillin in antimicrobial therapy in 1960, the emerging scope of drug-resistant incidents was significantly high. On the other hand, there is a void in new drug discovery for infections since 1990 till date. Though centrolineal approach was introduced in 2010, the mechanism was not a new one [2]. A timeline of various antibiotics introduced in the market is shown in Fig. 6. This void may be due to negligence on antimicrobial research or due to stringent regulatory guidelines on toxicity including tripartite ICH and OECD guidelines.

#### Essentiality of gene paradox

In the optimization of lead compound against drug resistance, the selected drug target or microbial protein should necessarily be valid and reliable in the resistant genome. Here, there is a challenge, despite the gene dispensability in resistant microbes or tumour cell and the possible potential targets; there is always a considerable amount of gene encoded proteins responsible for the unknown metabolic function. Hence, the full function of gene needs to be addressed in the validation process of a drug target. In practicality, it is not that so easy to build or define a chemical-based screening assay for the selected protein of unknown function [47, 48]. For example, 303 genes (7% of the *E. coli* genome) are found to be responsible for its growth in media (typical Waksman-screen conditions). Nevertheless, the nutrient deprivation and chemical perturbation have shown that further 258 genes are conditionally essential [49]. Thus, these 258 gene encoded enzymes are important for survival and its drug-resistant mechanism. Unfortunately, these types of investigations are ignored in the antibacterial drug discovery [50]. Still considerable efforts were made to reveal essential genes of model microorganisms through in vitro techniques; however, the essential genes responsible for the viability of resistant pathogens in infectious condition are only little understood [51].

#### Random transposon mutagenesis approach

Transposon mutagenesis known as transposition mutagenesis, it allows a gene to be transferred into the host organism’s chromosome that interrupts or modifies the function of an existing gene on the chromosome and cause mutation. This transposon-based mutagenesis is a powerful method to identify genetic elements that control specific phenotypes in antibacterial resistance. Essential genes can be assessed in drug-resistant pathogens using a random transposon mutagenesis approach [52]. This approach can assess a larger pool of mutants, which can be enabled by parallel DNA sequencing. This application was done for investigating the genomic requirements of *P. aeruginosa* in a sputum sample of patient with cystic fibrosis [53].

#### Interfering in vitamin biosynthesis of pathogens

Vitamin biosynthesis is a well-known process essential for survival of all pathogens, especially bacteria and fungi. The in vivo essential genes can represent the emerging targets, which are still unexplored in the current antibacterial and antifungal drug discovery [54]. For example, vitamin B2 (riboflavin) acts as a cofactor for ornithine-N^5^-monooxygenase SidA in *Aspergillus fumigates* by catalysing the first step in the biosynthesis of siderophores. Siderophores are known virulence factors which allow the fungi to overcome the iron deficiency [55]. Thus, the inhibition of riboflavin biosynthetic pathway in fungi reduces the formation of siderophores, which will inhibit the fungal iron acquisition and growth during infection. The combination of sulfamethoxazole and trimethoprim inhibit two key vitamin B9/folate biosynthetic enzymes of *Pneumocystis jirovecii* [56].

#### Drug screening in a non-conventional growth media

For the first time, this in vitro approach was employed for targeting the glyoxylate shunt of *P. aeruginosa* in pulmonary infection. This screening prioritizes the active compounds in nutrient-limited media where the media contains only acetate as a source of carbon. This screening procedure also prioritizes the inactive compounds only when glucose alone used as source. Supplementation of nutrient-limited media has proven to be a challenging mechanistic methodology to understand metabolic pathways and to investigate the mechanism of action of potent molecules [57]. Soon after the prioritization of the antibacterial lead, which was active in the absence of nutrient supplements, the systematic supplementation of individual and pools of metabolites to media is done to elucidate the mechanisms of action. There are reports on identified lead compounds which can target glycine, folate and biotin synthesis in *E. coli* [58].

#### Targeting the quorum-sensing virulence pathway

Quorum sensing is defined as the process of regulating the gene expression as a response to the change in cell population. It is very common in bacteria where the quorum-sensing bacteria release characteristic chemical signal molecules (auto inducers). The concentrations of auto inducers are directly proportional to the density of cell population. This approach was done on *P. aeruginosa* and has produced promising lead compounds. The resultant compounds were tested in animal models of induced infection without disturbing the in vitro growth [59].

#### Use of host model of disease

The macrophages are infected with *M. tuberculosis* and then the infected macrophages can identify the lead compounds. Usually, chemical compounds which interfere in respiratory mechanism like inhibitors of cytochrome bc1 complex may be discovered using this host model disease [60].

#### Genomic-chemical network

**Fig. 7:**
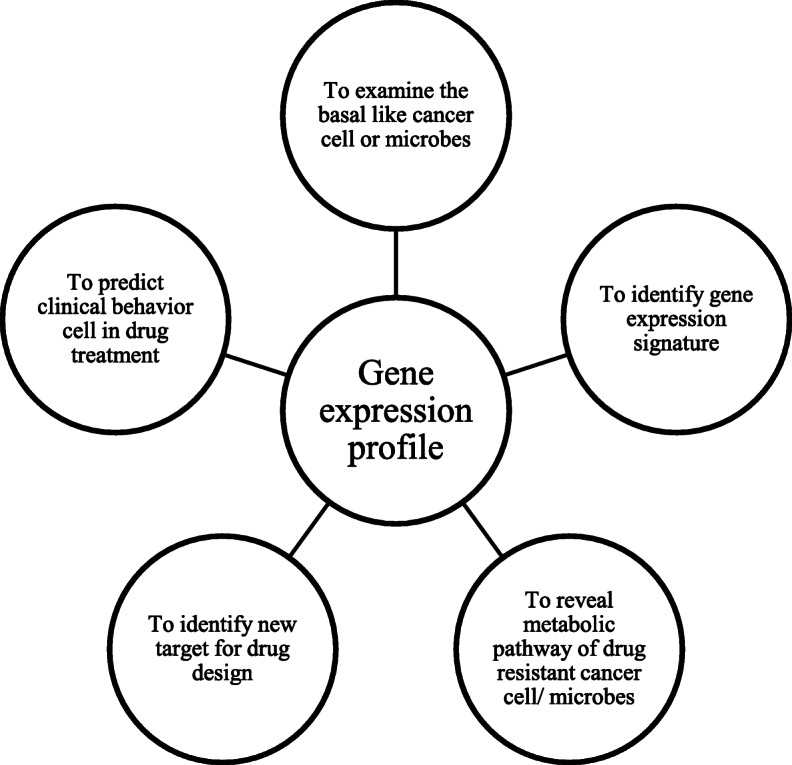
Importance of gene expression profiling in drug discovery against drug resistance

The existing drug discovery strategies have witnessed the transformation in the interpretation of the cell function based on genome. In yeast and bacteria, the investigation of interaction among genes and proteins has already established as a network of functional interactions. The classic mapping between cell metabolism and cell signalling are now displaced by cell network model. The cell network model characterizes a well-connected web of proteins and genes that are responsible for the complexity and redundancy [61]. This network helps in the study of synthetic lethality in the model organism to reveal the density of gene interactions and could serve as space for designing new drugs or combinations of drugs that probably inhibit the interacting gene. Indeed, this is a compelling case for a combinatorial approach, molecular docking and pharmacophore modelling to discover new antibiotics. There are reports available where the antibiotic sensitivity profile determined the *E. coli* gene knockout collection [62]. The overall importance of gene expression profiling in new drug discovery is represented in Fig. 7.

#### Synthetic viability and drug targets

For example, the synthetic viability is found in the biosynthetic pathway of the wall teichoic acid (WTA) of Gram-positive bacteria. The early steps in the biosynthetic pathway are dispensable, but the genes responsible for late-step enzymes are an essential phenotype. The essential phenotypes of the late genes are found to be due in the accumulation of biosynthetic intermediates of WTA and are linked to undecaprenyl moiety [61]. This undecaprenyl phosphate is acting as a lipid carrier in the process of peptidoglycan wall assembly. Thus, a unique dispensability pattern of WTA genes can serve as a basis for the discovery of new lead compounds [63].

#### Antibiotic adjuvants and combinatorial discovery

The antibiotic drugs possess dense and complex chemical-gene interaction network that resist perturbation. These networks provide a space for new targets for the design of antibiotic adjuvants. The antibiotic adjuvant is a non-antibiotic molecule that enhances the antimicrobial activity of antibiotics or it reduces the microbial resistance during treatment. The antimicrobial adjuvants have two major advantages in drug resistance viz increase the effectiveness of antimicrobials and reduce the occurrence of mutations. But this emerging complex network interaction of bacterial cell is quite challenging one for the modern genes-to-drugs approach and its ideology of target-validation measure [64]. The chemical-genomic characterization shall disclose the information and predictions about the pathogens mechanism of drug resistance. So if any lead compound discovered and characterized in this approach, it could be a ready probe for target validation and understanding of the network of the target. The suppressors are more commonly employed in drug-resistant tumours.

Previously, the combinations of antibiotic administration, such as trimethoprim plus sulfamethoxazole and amoxicillin plus β-lactamase inhibitor (Clavulanic acid), have been demonstrated as an effective strategy in the management of drug resistance. Even today, clinicians combine two classes of antibiotics such as β-lactams and aminoglycosides for achieving synergism if pathogens are unknown or to suppress the surfacing of drug resistance. Thus, the discovery of adjuvant combinations of two molecules would be more beneficial to reduce the incidence of resistance [3, 5].

Example, antibiotic efficacy of Novobiocin in *E. coli* was augmented by four new compounds that affect cell shape and membrane permeability [65]. Another example, aspergillomarasmine A (notorious carbapenemase NDM-1 blockers), reversed the carbapenem resistance in Gram-negative pathogens and *K. pneumonia-*infected mouse model. It was reported that loperamide induces destabilization of membrane potential in bacteria, which facilitates the increased permeability of minocycline, especially in Gram-negative bacteria [66]. In the same way, ticlopidine increases the efficacy of β-lactam antibiotics against MRSA through inhibition of the synthesis of WTA.

However, the combination approach can be consistent with the increased understanding of successful antibiotics mechanism if they bind with multiple numbers of targets [67]. Nevertheless, the modern target-based discovery approach devastatingly strives to identify the reliable target selectivity with minimized toxicity. Thus, the adjuvant approach adds an advantage to reuse the old and exhausted antibiotics in drug resistance era. But, the cause of unexpected drug–drug interactions must also be considered [68].

#### Chemical space vs. biological space

**Fig. 8:**
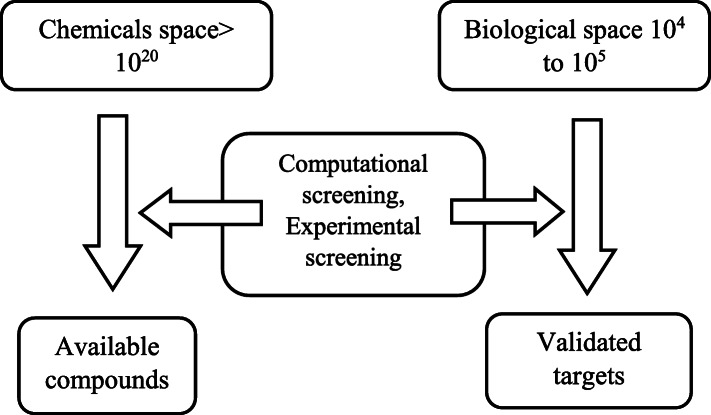
Role of biological–chemical space in drug target validation

To achieve the validated target in drug-resistant era, the gap between the compound library (more than 10^20^) and biological space (10^5^) is enormous as shown in Fig. 8. Thus, combination of computational, experimental screening and cellular genomic networks need to be employed for validating the drug target.

**Fig. 9:**
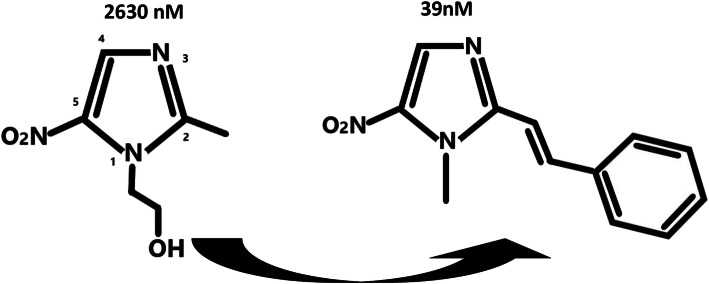
Example of structural diversity in potency—metronidazole and its potent derivative

These combinations may provide different fundamental techniques of modelling of biological proteins in the living cell system; it enables the researchers to find out effective drug strategies to solve the varying problems of AMR. These techniques also help in the analysis and interpretation of data like sequence of amino acid residues in proteins and interactions of protein ligand at the molecular level. Next challenge is that the integrative response between chemical structure and biology is not completely explored. Furthermore, the integrative response differs among different class of chemical compounds one from each other, despite the mechanism or drug target is similar or dissimilar. For example, modification of metronidazole at N^1^ and C^2^ positions improves the EC_50_ (against *Giardia lamblia)* to 39 nM from 2630 nM [67] shown in Fig. 9.

**Fig. 10:**
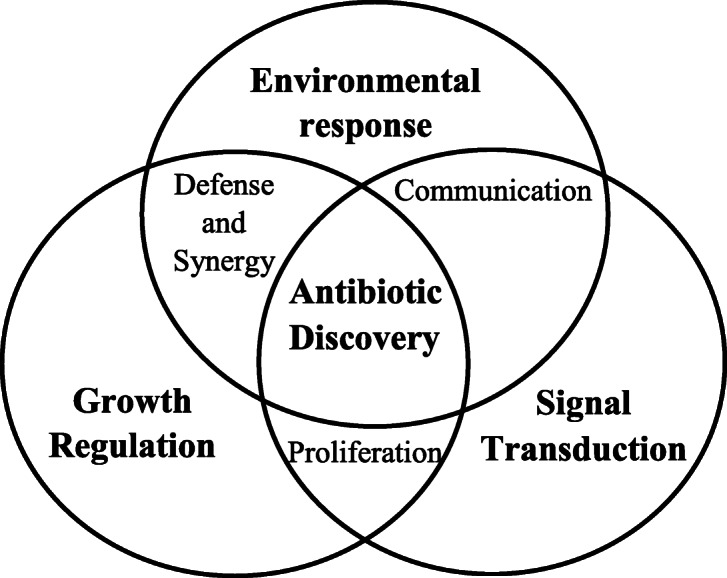
Strategies need for future antibiotic discovery

Although substantial development in the establishment of microbial genome has been taking place in recent time, yet the satisfactory outcome on cell biology of resistant microbes has to be much explored [69]. Thus, for future-generation antibiotics, the integrated knowledge among environmental response of microbes, growth regulation in mutants and signal transductions are needed to be explored, as represented in Fig. 10.

#### Identification of target by transcriptome profiling

In the field of molecular biology, gene expression profiling is the measurement of activity of thousands of genes (expression) at once, which is responsible for cellular function. The gene expression profiling is not new but more commonly used to differentiate normal cell from abnormal cell (tumour cell) or one type of cell from another with respect to (a) cell division; (b) response of cell to the environment and (c) response of cell to drug treatment.

In case of antimicrobial resistance, although several mutations in a pathogen are responsible for drug resistance, the relationships between mutations and phenotypic changes that are responsible for drug resistance not much explored. Also, it is noted that single mutation may cause many phenotype changes in organisms; thus, mutation-induced phenotype changes can cause both drug susceptibility and drug resistances [70].

The molecular mechanism of many drug-resistant microbes, parasites and cancer (especially breast cancer) with reference to biological or clinical behaviour is not well explored. In this, the microarray gene expression profiling would benefit us in many ways to find a solution as shown in the Fig. 7. The transcriptome profiling of antimicrobial resistance of *Pseudomonas aeruginosa* to fluoroquinolones and beta-lactam antibiotics was well established. Usually, the transcriptome profiling adapts the qualitative RNA sequencing to identify the genetic determinants [71]. It is believed that it can be better for next-generation sequencing (NGS). The NGS can accomplish (a) pathogen identification, (b) prompt initiation of target individualized treatment and (c) implementation of optimized control of drug resistance.

#### New antimicrobial molecules against drug resistance

The various drug discovery approaches have transformed drastically since 1925 (Table 5). The various approaches in drug discovery strategies are listed in Table 5. Newly identified molecules with novel mechanism of action in microbes are enlisted in Table 6. Subsequently the list of natural molecules with MIC values is reported in Table 7.

**Table 5:** Various timeline dependant drug discovery approaches and the expected success rate

Era	Year(s)	Approach	Success rate
Golden era	1950s	Natural product research Whole cell screening	High
Medicinal chemistry era	1975s	Synthetic tweaking Whole cell screening Broad spectrum	High
Resistant era	2000s	Modern drug discovery Target based Ligand based Broad spectrum	Low
Narrow-spectrum era	2025s	Unconventional discovery In vivo essential target Combinatorial approach Diagnostic development	Predicted

**Table 6:**
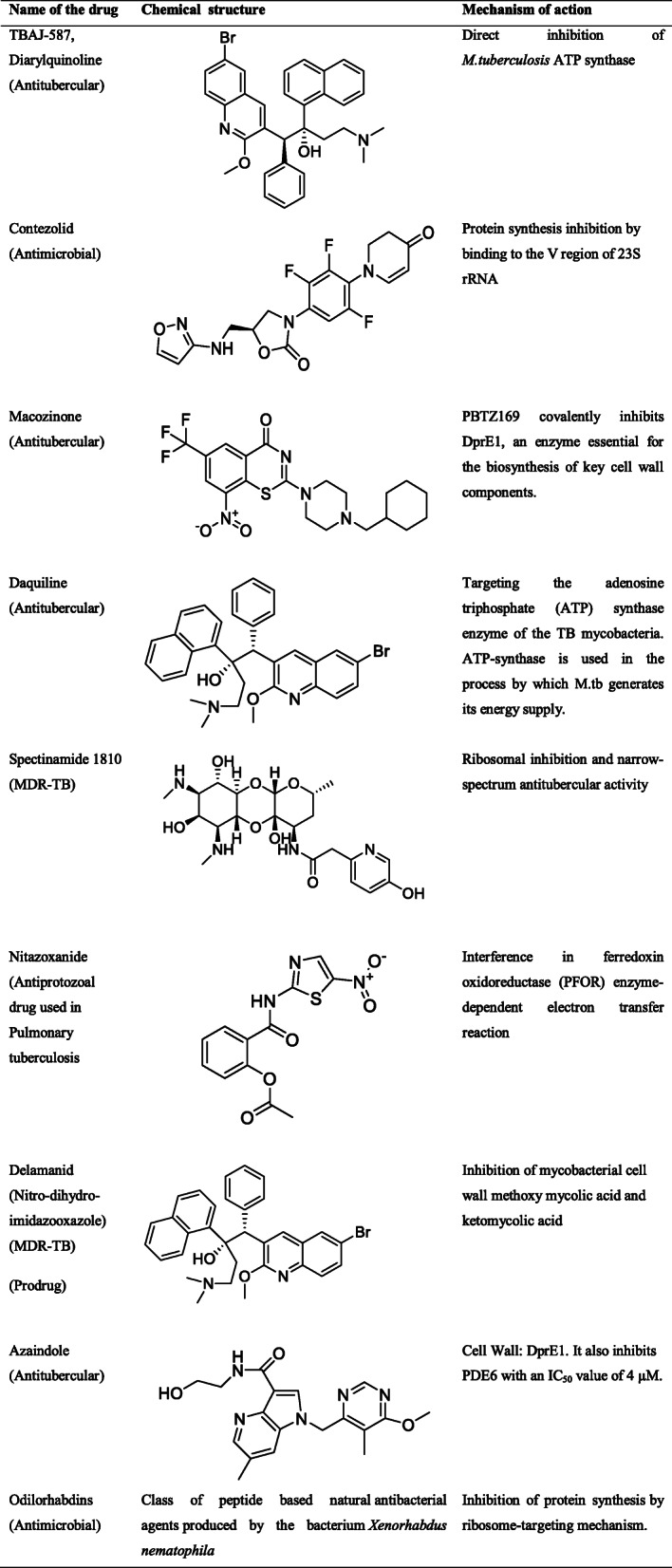
Newly identified potent molecules against drug-resistant infection with their reported mechanisms

**Table 7:**
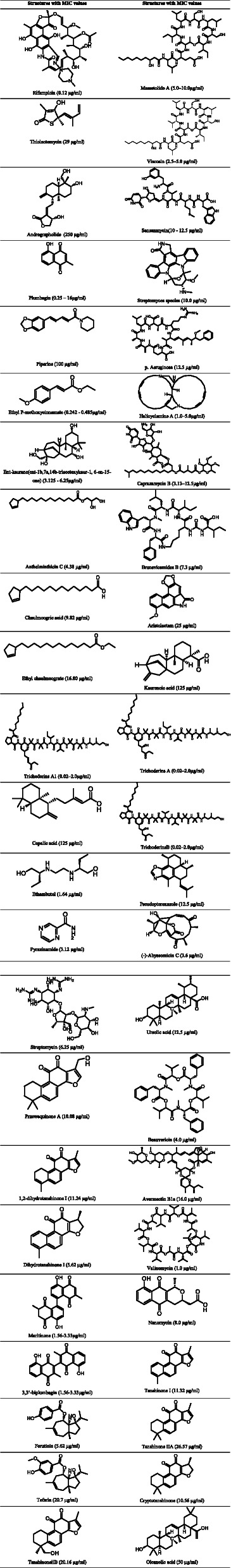
Reported natural compounds with considerable antitubercular activity with minimum inhibitory concentration in micrograms per millilitre

#### Photosensitization

There are three stages in killing pathogen by photosensitization. First, the photosensitization of drug-resistant pathogen is rationally potentiated with combination of visible light and using of inorganic salts (potassium iodide). Secondly, the use of blue and violet light is done to activate the photoactive porphyrins in bacteria. Lastly, the safe UV rays at a wavelength range from 200 to 230 nm are used to kill microbial cells without damaging host mammalian cells. The evidence from in vitro studies has been established that the photosensitization can kill multidrug-resistant bacteria and they do not develop any resistance to UV light. Hence, there are animal models for localized infections caused by resistant species that are monitored by non-invasive bioluminescence imaging (BLI). Bioluminescence imaging (BLI) is a technique that reports activity at the molecular level using light produced by enzyme-catalysed reactions. For non-invasive imaging in cell biology and small animal studies, bioluminescent reporters need a small chemical substrate. Incident light such as fluorescence or phosphorescence is not necessary for BLI, preventing photo toxicity [72]. The disadvantages of photosensitivity are (a) photosensitization after treatment, (b) the efficacy of the tumour treatment influenced by the precise delivery of the light, (c) oxygenation of tissue is vital by the photodynamic effect, (d) current photosensitivity methods are not expedient to treat metastatic cancers [73].

#### Antimicrobial or host defence peptide

Antimicrobial peptides (AMPs) are also known as host defence peptides (HDPs). These peptides are found in all living beings as a part of innate immune response. The exploration of the differences exists between prokaryotic and eukaryotic cells with respect to HDPs and may provide the idea for targeting the antimicrobial peptides. Peptide-based antibiotics are relatively small molecules, and unlike classical antibiotics, they act very fast and are broad spectrum; thus, they are lethal to several types of pathogens. Further, interestingly, they seem to be free from several drug resistance mechanisms of microbes. The distinct advantage of peptides over classical antibiotics is they portray a highly modular synthetic antimicrobial system and they kill microbes by destroying their membrane. In addition to antibiotics activity, these peptides can also inhibit enveloped viruses, fungi and cancerous cells [74]. These peptides are also acting as immunomodulators (examples of AMPs are dermicidin (cationic human peptide), cecropins, andropin, moricin, ceratotoxin, melittin (cationic alpha-helical insect peptide), magainin, dermaseptin, bombinin, brevinin-1, esculentins and buforin II (amphibians), indolicidin (cattle) and defensins (anionic human peptide)). The AMP drugs approved by FDA are bacitracin, dalbavancin, daptomycin, enfuvirtide, oritavancin, teicoplanin, telaprevir, telavancin, vancomycin, etc. [75]. Nearly 36 AMPs are in clinical trials (clinical/preclinical), among these MU1140, D2A21, HB1275, HB1345, arenicin, AP114, AP138, novamycin, novarifyn, avidocin and purocin were in preclinical stage; NVB-302 and friulimicin B were in phase I studies; EA-230, CZEN-002, delmitide, ghrelin, hLF1-11, Wap-8294A2, C16G2, DPK-060, PAC113, LTX-109, OP-145, LL-37 and novexatin were in phase II studies; and D2A21, XMP-629, neuprex, delmitide, ghrelin, SGX942, PXL01, POL7080, p2TA, iseganan, pexiganan, omiganan, surotomycin and ramoplanin were in phase III [76].

### Antievolution drugs

Despite the mechanisms of antibiotic-induced mutagenesis in microbes, the need of understanding the underlying molecular mechanism of evolutionary resistance in pathogenic bacteria against our immune systems and antibiotics is very essential. Recently, in 2019, John et al. found that ciprofloxacin induced the mutation via reactive oxygen species (ROS) in *E. coli*. The ROS can enhance the stress environment by triggering the activation of *Escherichia coli* SOS DNA-damage response and error-prone DNA polymerases in all cells. Hence, the FDA approved the drug ‘edaravone’, administered along with antibiotic which resulted in decreased levels of ROS in *E. coli* population; thus, edaravone can down the mutations in the bacteria [77].

### Nanotheranostics

Nanotheranostics are the novel drug delivery approach based on the integration of both diagnostic and therapeutic function in a unit drug delivery system, which is now extremely attracted for personalized medicine. Previously, there was very limited drug delivery approach to fight drug resistance in microbes and cancer.

#### Antimicrobial nanotheranostics

In recent time, a number of nanoparticle (NP) drug delivery approaches, including nanotheranostics, have been investigated and reported [78]. They revealed the antimicrobial efficacy of nanoparticles including silver NP, gold NP, bimetallic NP, copper oxide NP, iron oxide NP, zinc oxide NP, etc. In addition, liposomal nano formulations were also reported for antibiotics including amikacin and ciprofloxacin which are under clinical trials. Though the silver nanoparticles (AgNPs) have been known for broad-spectrum antibacterial property against drug-resistant bacteria, the mechanism was elusive. But in a recent study, the light-excited AgNPs induced protein aggregation in *E. coli*, which relied on the light-catalysed oxidation of cellular proteins. It seems that AgNPs can absorb the photon energy and can transfer energy to the bacterial proteins, thus promoting the bacterial protein to undergo degradation and leading to death. Furthermore, the isobaric tags for relative and absolute quantification (iTRAQ)-based proteomics showed that the bacteria had failed to develop resistance to the light-excited nanoparticles [79].

#### Anticancer nanotheranostics

The co-encapsulated nanoparticles containing daunorubicin and glycyrrhizic acid significantly inhibited the growth of drug-resistant leukaemia cells and bypassed the drug resistance. It was also reported that the formulated nanoparticles enhanced the drug uptake in the resistant K562/A02 cells. Furthermore, the modified P-glycoprotein antibody on the nanoparticles has further increased the drug uptake in the leukaemia cells [80]. Still there are challenges in nanoparticle delivery to tumour cells, and they include the need for assessing the interactions of nano-antibiotics with cells, tissues and organs for dose optimization and for appropriate routes of drug delivery [81]. The biocompatibility of NPs need to be evaluated because NPs can enter through skin contact, ingestion, inhalation, oral and intravenous injection; hence, the appropriate in vivo models need to be used to understand the insights on their potential toxicity and metabolism [82].

### New drug targets and approaches

The outcome of new antimicrobial drug with the conventional drug discovery approach is very slow and the future outcomes are also not optimistic. Hence, there is a need for reanalyzing the discovery strategy as integrative approach towards achieving new class of antibiotics and new antimicrobial adjuvants, but focusing on alternative validated targets [20, 21, 83]. Thus, antimicrobial adjuvants [84, 85] act as (a) efflux pumps inhibitors, (b) permeability enhancers, (c) virulence factor inhibitions, (d) transfer inhibitions and (e) anti-quorum sensing. The possible antimicrobial drug targets are discussed below.

#### NagZ Protein

It is a cytoplasm protein responsible for development of drug resistance in microbes through beta-lactamase expression, cell recycling process and biofilm formation. The NagZ protein is known as *N*-acetyl-β-d-glucosaminidase and plays a crucial role in the peptidoglycan recycling pathway of Gram-negative bacteria [86]. The product of NagZ protein, 1,6-anhydromuramoyl-peptide, is responsible for the induction β-lactam resistance in many bacteria and is induced by the expression of AmpC β-lactamase [87]. Hence, inhibitors of NagZ activity like PUGNAc, MM-124 (non-selective), EtBuPUG and MM-156 (selective) could suppress β-lactam antibiotic resistance in bacteria [88, 89].

#### AmpG Protein

**Fig. 11:**
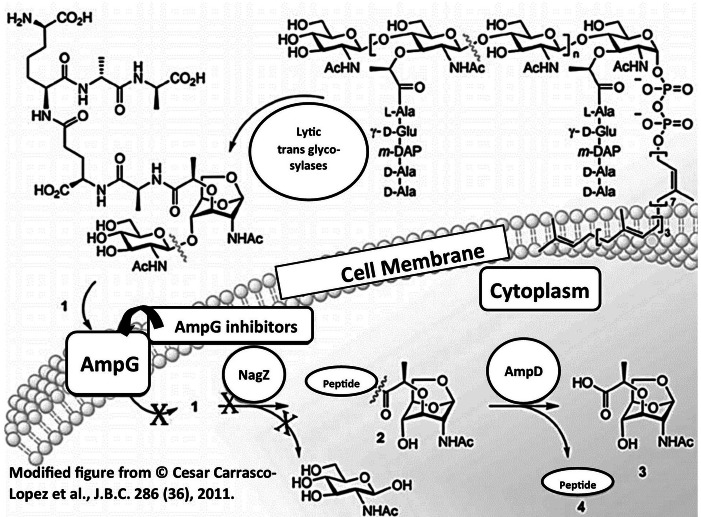
Proposed mechanism for AmpG inhibitors which interfere in intracellular protein synthesis

It is only the permease class of protein present in the cell membrane of drug-resistant microbes and acting as signal transducer for beta-lactamase production and internalization of soluble muropeptide into cytoplasm. Other proteins such as AmpC and AmpD are present in the cytoplasm and they are involved only in the cell recycling process. Inhibition of AmpG will lead to depletion of beta-lactamase enzyme; thus, the design of AmpG inhibitors (fosfomycin) could be a better choice to suppress the release of beta-lactamase in microbes, and these agents can serve as more suitable antimicrobial adjuvants to β-lactam antibiotics [90]. In addition, the inhibition of AmpG proteins will lead to the termination of the cell wall recycling process; thus, the permeability of the cell wall can also be greatly enhanced. Hence, these agents could also serve as permeability enhancer to all potent antibiotics/fluoroquinolones [91]. The AmpG may be the more suitable drug target due to the proven mechanism of beta-lactamase expression in many drug-resistant bacteria and more reliable for its location (cell membrane) as compared to NagZ (cytoplasm), shown in Fig. 11.

#### Polyphosphate kinase

In bacteria, the synthesis of inorganic polyphosphate (Poly P) from ATP is catalysed by polyphosphate kinase (PPK). Three polyphosphate kinase enzymes including PPK1, PPK2 and PPK3 have been documented as responsible marker for the accumulation of inorganic polyphosphate in microbes including *Mycobacterium tuberculosis* through animal models [92]. Among all, the role of PPK2 has been well studied in the development of drug resistance, virulence and cell wall permeability to polar TB drugs. Hence, it would be beneficial to design a new small molecule as PPK2 inhibitors (NSC 35676, NSC 30205, NSC 345647 and NSC 9037) as adjuvant to the existing antibiotics and antitubercular drugs [93]. There are reports on small molecules of PPK2 inhibitors, increased antitubercular activity of isoniazid (8 fold) and polar drugs. Therefore, adding these PPK2 inhibitors as adjuvant to the existing anti-TB drugs or antibiotics would benefit the existing drug regimen for the cure of drug-resistant infections [94, 95].

#### Cytochrome bc1 complex

**Fig. 12:**
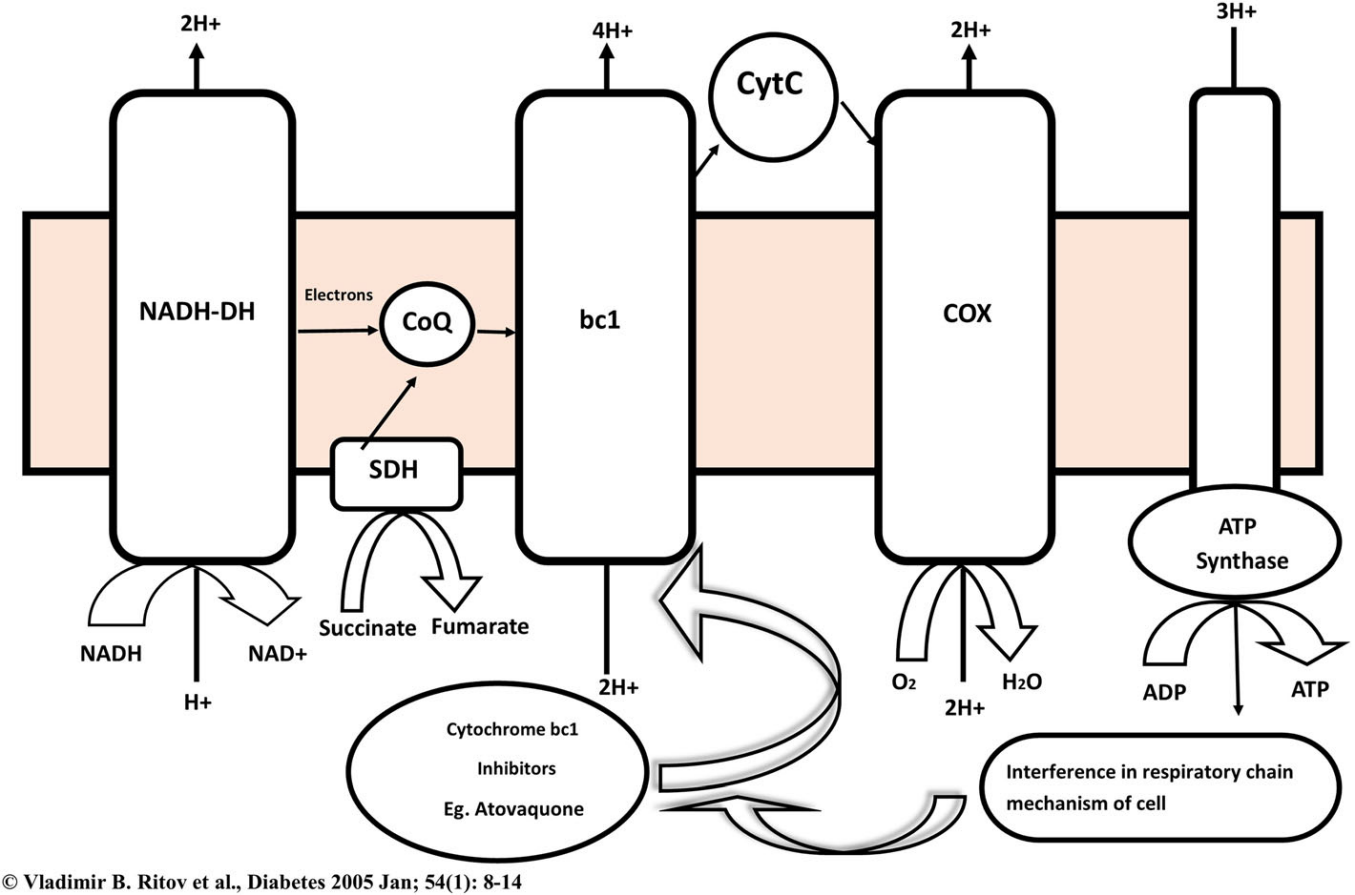
Proposed mechanism for cytochrome bc1 inhibition which result in inhibition of ATP synthesis

In all microbes, cytochrome bc1 complex (complex III) is responsible for mitochondrial respiratory chain in the intracellular metabolic pathways. Thus inhibition of cytochrome bc1 complex will lead to the depletion of ATP and results in bactericidal action. Several experts quoted that the cytochrome bc1 complex could be the most suitable target for MDR/XDR-TB among complex I to V. Complex III (Cytochrome bc1, Cytochrome c) is playing a critical role in the biochemical generation of ATP in Fig. 12. There are reports on inhibitors of cytochrome bc1 at Q site [96], e.g. lansoprazole [97], atovaquone [98, 99], antimycin [100], stigmatellin [101] and myxothiazole [102], where these inhibitors induced lethal effect to mycobacterium, by inhibiting electron transfer form Cyt b to Cyt c. Furthermore, there is no evidence for acquired mutation associated with mycobacterial cytochrome bc1 complex.

#### Auto-inducers (AIs)

Auto-inducers are signalling molecules produced in microbial culture as a response to the change in microbial population density. Therefore, the density of quorum-sensing bacterial cells is directly proportional to the concentration of the auto-inducers. Quorum sensing is the ability of bacteria to sense one another, which exists in both Gram-negative and Gram-positive bacteria. Through this quorum sensing, bacteria regulate variety of activities including symbiosis, virulence, motility, antibiotic production and biofilm formation [103].

Auto-inducers differ from species to species and allow the microbes to communicate within and between different species [104]. Three major types of auto-inducers (AIs) exist in most of the Gram-negative organisms, e.g. 4-nitro-pyridine-N-oxide (4-NPO), *Acylated homoserine lactones* (AHL), N-3-oxohexanoyl-L-homoserine lactones (AI-1).

*Auto-inducing peptides* (AIPs)

AIPs found in Gram-positive organisms are processed, modified and excreted by amino acids or short peptides synthesized/processed/modified/excreted by the ATP-binding cassette export systems. AIP binds to the cell surface-bound histidine protein kinase that auto-phosphorylates which is a response regulator responsible for the activation of transcription of target genes.

*Auto-inducer-2 compounds*

AI-2 are derived furanones present in both Gram-negative and Gram-positive bacteria. As an example, LuxS (enzyme) produces 4, 5-dihydroxy-2, 3-pentanedione (DPD) acylhomoserine lactones which are a forerunner of AI-2. This auto-inducer binds to LuxP protein to produce AI-2/LuxP complex. The complex binds to the membrane-bound histidine protein kinase. Further, signal transduction channels in multistep phosphorylation are similar to AIPs [105].

#### LuxS and Quorum-sensing inhibitors (QSIs)

They are derived from the information of AIs. QSIs act through the blocking of signal synthesis [106]. For example, LuxS is the target, and the inhibitors of LuxS could act as broad-spectrum antibiotics [107]. The other possible blockade strategies are targeting the auto-inducer receptor site of the LuxR homologues, histidine protein kinase or Lsr transporter [108]. Additional approach for QSIs is to block the formation of active dimers (essential for binding and expression of target genes and enhancement of signal molecule degradation) [83]. Always the combination of both mechanisms will be more effective than a single mechanism approach. The reported molecules as QS inhibitors (QSI) are agrocinopine B, furanone, Canavanine, norepinephrine, epinephrine, penicillic acid and patulin.

#### TCST system

The two-component signal transduction (TCST) systems are the primary means for coordinating responses to environmental changes in bacterium as similar to some plants, fungi, protozoa and archaea [106]. These systems generally comprise a receptor essential amino acid enzyme, histidine kinase (HK), that will react to an extracellular signal by phosphorylating cytoplasmic response regulator [109]. The most common inhibitors reportable so far are hydrophobic compounds that inhibit HK-autokinase activity, noncompetitively with relevance to ATP 11 [110, 111].

#### Division machinery targets

The division machinery of bacteria can be a striking drug target, and seven or additional essential proteins are preserved virtually throughout the bacterial kingdom; however, these proteins are absent in humans. Examples, the polymerized filamenting temperature-sensitive mutant Z (FtsZ) recruits alternative cell proteins, together with FtsA, ZipA, FtsK, FtsQ, FtsL, FtsW, FtsI and FtsN, resulting in the formation of a Z-ring and also the initiation of the complicated method of partitioning [105].

#### 1-Deoxy-d-xylulose-5-phosphate (DOXP) synthase

The enzyme, 1-deoxy-d-xylulose-5-phosphate (DOXP) synthase is known to be inhibited by fosmidomycin (antibiotic from gram-positive bacterium). This enzyme is also profound in alternative, non-mevalonate pathway for the production of carotenoids, phytol, plastoquinone-9, isoprene and mono- and diterpenes [106]. Now, the enzymes of the 1-deoxy-d-xylulose 5-phosphate (DOXP) and 2-C-methylerythritol 4-phosphate (MEP) pathway were identified as targets for new herbicides and antibacterial drugs. Till today, no inhibitors for the DOXP synthase have been discovered. There are reports where clomazone degradation products showed inhibition on DOXP synthase [108].

#### Enzymes of fatty acid synthesis

FabH, FAabG, FAbI and FabF/B are essential enzymes in fatty acid (type II pathway) synthesis in microbes. They are extremely enticing targets for the development of antibacterial and antiparasitic compounds [108]. Two natural products specifically cerulenin and thiolactomycin inhibited the compressing or condensation enzymes FabH and FabF/B. Cerulenin shows selectivity on FabF/B, whereas thiolactomycin (TLM) and its analogues shows inhibition on FabH and FabF/B. Acyl group carrier protein synthesis (AcpS) is very important in the fatty synthesis in true bacterium or Mycobacterium. CoaD isozyme has additionally gained much importance in the medicinal drug design or antibacterial drug design target [109].

#### MUR inhibitors

The MurA (UDP-N-acetylglucosamine enolpyruvyl transferase) is a fundamental enzyme found in bacteria, responsible for transferring enolpyruvate (EP) from phosphoenolpyruvate (PEP) to uridine diphosphate N-acetylglucosamine (UDP-GlcNAc). This step is the first biochemical in bacterial peptidoglycan synthesis. This MurA is conserved in bacteria, but not in human. Hence, this enzyme can be a drug target for antibacterial drug discovery. Furthermore, this target has been validated using fosfomycin (RWJ-3981, RWJ-110192 and RWJ-140998 are in trials) which is currently available in clinical use. This fosfomycin shows covalent binding to Cys115 in the MurA enzyme and thus interrupts the active site responsible for release of UDP-GlcNAc-enoylpyruvate and ultimately leading to cell death [112].

#### Combination therapy and antibiotic-free treatment to tackle drug resistance

In 2018, researchers at the University of California, Los Angeles (UCLA) stated that combining four or five antibiotics stopped or slowed down the severity of drug-resistant bacterial infections. The team used a mathematical model called as MAGIC (mathematical analysis of general interactions of the components), which has enabled them to anticipate their results of antibiotic combination and the team suggested about 8000 theoretical combinations [113]. A new animal experiment has shown the efficacy of bacterial toxin-grabbing nanoparticles (Dutch biotech company), which involved the use of machinery from phages (bacteria-killing virus) to target the drug-resistant *Staphylococcus aureus*.

## Conclusion

Overall, the amount of advancement in research to curtail the antimicrobial resistance is quite satisfactory. The various advancements in drug discovery are gene paradox, transposon mutagenesis, interfering vitamin and fatty acid biosynthesis, use of non-conventional growth media and host model for drug screening and targeting the quorum-sensing virulence pathway. Many researchers emphasized that drug target of resistant microbes which are essential in fatty acid biosynthesis, vitamin biosynthesis and respiratory mechanism in microbes would be more beneficial. In the literature, we found that there has been lot of natural hit molecules which are structurally diverged and have proven their acceptable antimicrobial activity on virulent species including *Mycobacterium tuberculosis*. These molecules have to be further investigated to enhance their antimicrobial efficacy in drug-resistant microbes or to assess their suitability as co-administered agent with standard regimen. It is highly recommended to retrieve the life of existing antibiotics through antimicrobial adjuvant discovery. Hence, it is highly recommended to execute the anti-drug resistance research as integrated approach where both molecular and genetic research needs to be the integrative objective of drug discovery. The hope for new hit drugs to fight drug resistance is not optimistic due to existing discovery void between genetic and molecule research and lack of validated drug targets. Overall, we conclude that this is time to accelerate new drug discovery research with advanced genetic approaches instead of conventional blind screening.

## Data Availability

NA

## Abbreviations

MDR: Multidrug resistance
XDR: Extensively drug-resistant
MRSA: Methicillin-resistant *Staphylococcus aureus*
VRSA: Vancomycin-resistant *Staphylococcus aureus*
CRE: Carbapenem-resistant Enterobacteriaceae
HSCT: Hematopoietic stem cell transplant
ABC: ATP-binding cassette
MFS: Major facilitator super-family
OMP: Outer membrane protein
ACTs: Artemisinin combinational therapies
PfCRT: *P. falciparum* chloroquine-resistant transporter
oAMT: Oral artemisinin-based monotherapy
IAV: Influenza A virus
HSV: Herpes simplex virus
HCMV: Human cytomegalovirus
HBV: Hepatitis B virus
HCV: Hepatitis C virus
HA: Hemagglutinin
NA: Neuraminidase
WTA: Wall teichoic acid
NGS: Next-generation sequencing
AMPs: Antimicrobial peptides
HDPs: Host defence peptides
ROS: Reactive oxygen species
iTRAQ: Isobaric tags for relative and absolute quantification
PPK: Polyphosphate kinase
AHL: Acylatedhomoserine lactones
AIPs: Auto-inducing peptides
QSIs: Quorum-sensing inhibitors
TCST: Two-component signal transduction
HK: Histidine kinase
FtsZ: Filamenting temperature-sensitive mutant Z
DOXP: 1-Deoxy-d-xylulose-5-phosphate
MEP: 2-C-methylerythritol 4-phosphate
TLM: Thiolactomycin
AcpS: Acyl group carrier protein synthesis
EP: Enolpyruvate
